# Novel treatment of chalazion using light-guided-tip intense pulsed light

**DOI:** 10.1038/s41598-023-39332-x

**Published:** 2023-07-31

**Authors:** Yirui Zhu, Hanhe Zhao, Xiaodan Huang, Lin Lin, Yanan Huo, Zhenwei Qin, Jiale Lu, Xiuming Jin

**Affiliations:** 1 Eye Center, School of Medicine, The Second Affiliated Hospital, Zhejiang University, 88 Jiefang Road, Hangzhou, 310009 Zhejiang China; 2 Zhejiang Provincial Key Laboratory of Ophthalmology, Hangzhou, Zhejiang China; 3 Zhejiang Provincial Clinical Research Center for Eye Diseases, Zhejiang Provincial Engineering Institute on Eye Diseases, Hangzhou, Zhejiang China

**Keywords:** Medical research, Clinical trial design, Clinical trials

## Abstract

We assessed the effectiveness of light-guided-tip intense pulsed light (IPL) with meibomian gland expression (MGX) in chalazion treatment. Ninety-five eyes with chalazion received a light-guided-tip IPL-MGX treatment (IPL-MGX group), and another 95 eyes with chalazion received incision with curettage treatment (Control group). Prior to IPL or incision, as well as 1 month after the final treatment, data were gathered pertaining to the lesion location and size, hyperemia, lesions regression or recurrence, and a comprehensive ophthalmic examination. The total size of the chalazia in the IPL-MGX group was significantly reduced after the final treatment, with an average resolution rate of 70.5%, which is comparable to excision surgery. A significant decrease in chalazion recurrence rate was apparent after treatment in the IPL-MGX group compared with control. Moreover, the IPL-MGX demonstrated significant advancements throughout noninvasive tear film breakup time (NIBUT) as well as meibum grade in comparison to baseline and those in the the Control group. The use of IPL-MGX was found to be an efficient therapy for reducing the size and recurring frequency of chalazia, as well as for improving the meibomian gland function. It may be considered as a first-line treatment for cases of primary or recurrent chalazia with inflammation.

## Introduction

A chalazion is an acute lipogranulomatous inflammation that is usually caused by plugged meibomian gland ducts and frequently becomes chronic^1^. Individuals of all ages, including children, are susceptible to this most common eyelid disease^2^. Earlier research has shown that patients who suffer from meibomian gland dysfunction (MGD) and chronic blepharokeratoconjunctivitis have a greater chalazion incidence as well as a recurrence frequency. This is due to the fact that these patients have suffered from poor meibomian gland function for an extended period of time, which resultantly changes the glands morphological characteristics^3^.

In 25% to 50% of cases, chalazion is self-limiting with the ability to be resolved or enhanced by medical therapy throughout 1–3 months of onset^4^. A variety of treatment methods are available, including eyelid hygiene with warm compresses, antibiotic ophthalmic ointment, steroid injection, lesion excision with curettage, and total excision^5^. The treatments of excision and curettage are believed to be straightforward and successful; however, they can produce discomfort in the eyelid. A primary and recurrent chalazion may cause cosmetic disfigurement to the eyelid, ocular inflammation together with irritation, and possibly even visual impairment due to mechanical ptosis and corneal astigmatism^6^. Patients with recurrent and refractory chalazia have increased risks of depression, anxiety, and decreased quality of life. In our previous study, we reported high rates of resistant and recurring chalaziosis following therapeutic therapy including conservative therapy and lesion excision with curettage^7^. We found that the combination of IPL treatment and MGX offers a low risk and effective option in decreasing the recurrence rate of recurrent chalaziosis post excision by improving meibomian gland function. Therefore, focusing on the functional and anatomical restoration of the meibomian gland by IPL, not just by traditional therapy such as lesion excision with curettage. Moreover, a new noninvasive treatment is essential to resolve chalazia and improve meibomian gland function.

Intense pulsed light (IPL) has been widely adopted in the cosmetic industry and therapeutically to remove facial telangiectasia and rosacea, benign cavernous hemangiomas, venous malformations, and pigmented lesions^8,9^. An improvement in ocular surface health was observed in individuals who underwent IPL therapy for the dermatologic manifestations of rosacea^10^. Ophthalmologists have demonstrated the efficacy and safety of IPL and meibomian gland expression (MGX) treatment in improving dry eye symptoms and MGD^10–12^.

Recently, IPL applied with a 6 mm cylindrical light-guided-tip to the upper eyelids was found to improve dry eye symptoms^13^. This study suggests that light-guided-tip IPL treatment is safe and effective for patients with dry eye symptoms and visible signs of MGD. In a prior study, we analyzed the difference in the chalaziosis recurrence rate following excision and curettage operation with or without IPL-MGX treatment^7^. The results showed that following surgery, patients who had IPL-MGX therapy saw a substantial improvement in meibomian gland function and a reduction in chalazion recurrence. IPL warms the skin internally to 50 °C helps liquefy aberrant meibum and assists telangiectasias to absorb more energy to closure abnormal vessels^14,15^. This has led to the interest in IPL as a potential therapy for chalazion.

To date, few studies have described the resolution and recurrence rates of light-guided-tip IPL for chalazion. Arita et al. reported the efficacy and safety of IPL on cheeks and eyelids combined with MGX for the treatment of refractory multiple and recurrent chalazia without surgery or curettage^16^. This research evaluated a novel treatment of the light-guided-tip IPL-MGX effectiveness and safety in primary or recurring chalazion.

## Patients and methods

This was a controlled, prospective, open-label clinical experiment. It was authorized by the hospital's institutional review board and recorded in the Clinical Trial Registry (NCT05512572) in 23/08/2022. The research was done from September 2021 to March 2022 at the Eye Clinic of Zhejiang University's Second Affiliated Hospital. Before participating, participants gave written permission. This study was approved by the Institutional Review Board of the Affiliated Second Hospital of Zhejiang University. The protocol adhered to the tenets of the Declaration of Helsinki. Written informed consent was obtained from all subjects.

### Subjects

Participants were chalazion patients who visited the Eye Clinic and met the following criteria: (1) aged 18 at least, without gender restriction; (2) Patients with chalazion in the inflammatory phase (defined by the contents of the glands are released into the tarsus and the surrounding eyelid soft tissue, eliciting an acute inflammatory response accompanied by pain and erythema^17^ on slit-lamp examination with symptoms who did not respond to conservative therapy, including antibiotic ophthalmic ointments and warm compresses for 1 week; (3) sun sensitive Fitzpatrick skin types 1–4 patients^18^ who didn’t have skin cancer or other conditions preventing IPL therapy; and (4) patients who choose to undergo IPL-MGX therapy (IPL-MGX group).

Age-, sex- and diagnosis-matched consecutive patients with chalazion who failed to respond to antibiotic ophthalmic ointments and warm compresses and received incision with curettage but without IPL-MGX treatment were included as controls (Control group).

Exclusions were based on the following criteria: (1) any ocular disease or surgery, such as ocular infection, allergy, intraocular inflammation, trauma throughout the previous 6 months; (2) any eyelid disorder; (3) any systemic condition that may cause dry eye; (4) any skin disease or pigmented lesion in the treatment zone; (5) pregnancy or lactation; and (6) any other condition that could affect the ocular surface and could make the patient inappropriate for inclusion in the study.

### Experimental design

**Figure 1:**
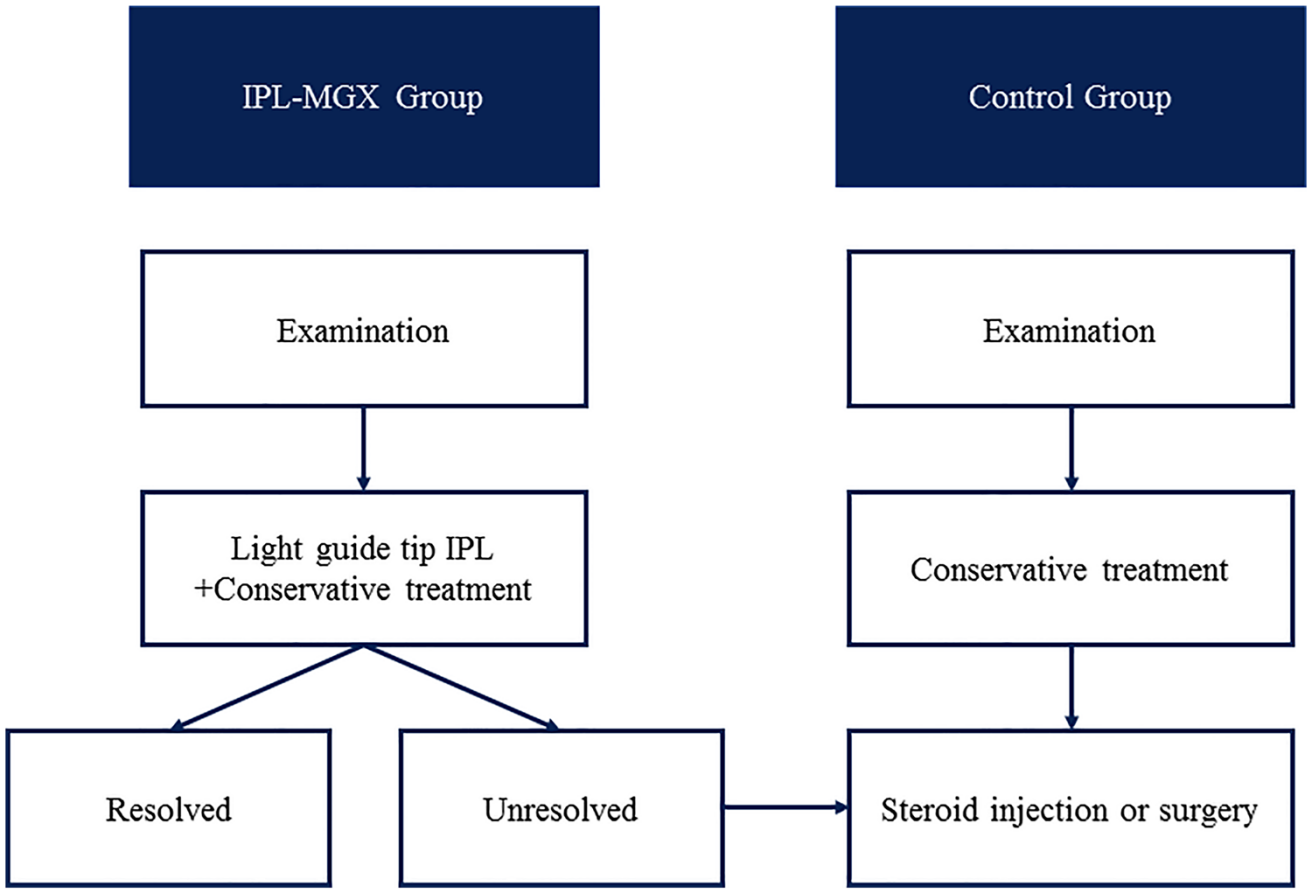
The treatment protocol and follow-up schedule for the IPL-MGX and Control groups are presented.

Each IPL-MGX patient completed a series of IPL-MGX treatment sessions at 3-week intervals depending on the chalazion to be resolved. The IPL was terminated when the size of chalazion was reduced by 80–100%. A minimum 80% reduction in chalazion size without recurring was considered a resolution. After IPL treatment, patients were continued to be offered antibiotic ointments and/or warm compresses treatment. Each control patient who failed to respond to conservative therapy received incision with curettage but without IPL-MGX treatment. Each patient underwent clinical assessment before treatment and at 1 month after the final treatment. All the patients continued to apply warm compresses three times a day, levofloxacin eyedrops (Santen, Osaka, Japan) four times a day, and tobramycin and dexamethasone eye ointment (Alcon Laboratories, Fort Worth, TX) to the eyelids twice a day during the study. No patient was permitted to initiate topical or systemic agents for chalazion or MGD during treatment. The efficacy outcome measures of chalazion recurrence were screened at 6 months after treatment (Fig. 1).

### IPL-MGX treatment

**Figure 2:**
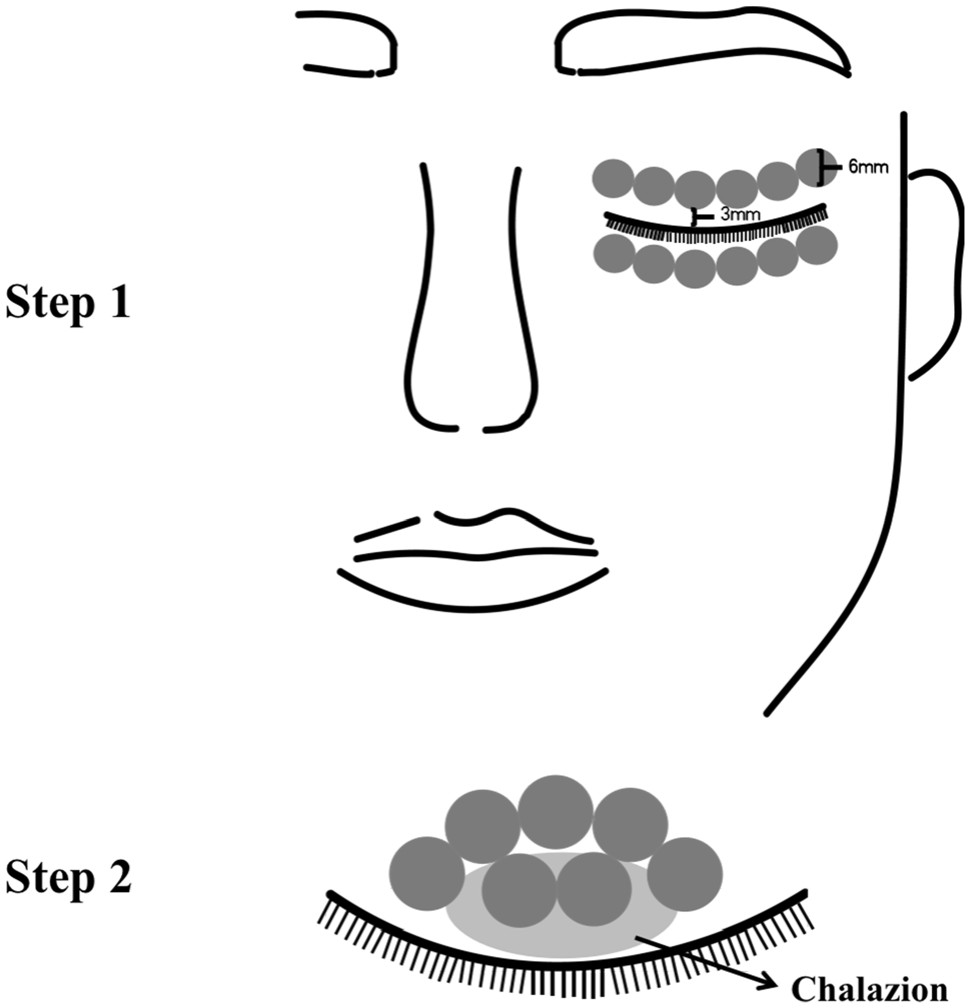
(step 1) Light guide tip IPL treatment zone including six overlapping periocular areas (6 mm diameter each) on each eyelid. (step 2) More 6–10 pulses of IPL were applied around chalazion area with slightly overlapping.

**Figure 3:**
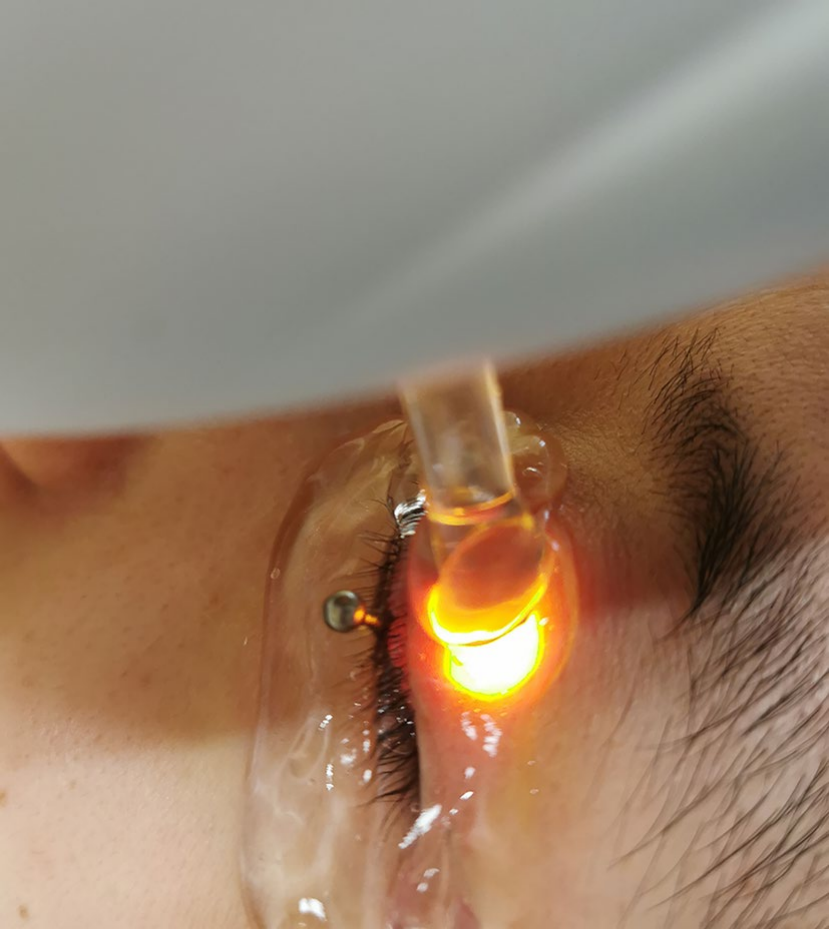
Protection of the cornea and sclera with the IPL-aid eye shield in the conjunctival sac during IPL treatment.

This research used an IPL system with optimum pulse technology (M22; Lumenis, Yokneam, Israel) consisting of a 515–1200 nm xenon lamp and a 590-nm filter. Before the initial IPL treatment, patients' Fitzpatrick skin types were determined, and the IPL machine was set at the proper setting (12–14 J/cm^2^). After removing face and eyelid oil, a topical anesthetic was administered (compound lidocaine cream; Ziguang Pharmaceutical Co. Ltd., Beijing, China). Thirty minutes later, the cream wiped off, following topically administered proparacaine eye drops. The cornea as well as sclera of treated eye were fully protected with a sterile IPL-aid eye shield applied on the conjunctival sac (Suzhou Mingren Medical Equipment Co. Ltd., Suzhou, China). An ultrasound gel coating was placed to the upper and lower eyelid regions to be treated. In step 1, upper and lower eyelid periocular regions of treated eye received 12 IPL pulses. In step 2, 6 to 10 IPL pulses were administered around each chalazion using a light-guided-tip (6-mm-diameter cylindrical handpiece, M22; Lumenis, Yokneam, Israel). The IPL pulses were applied 2–3 mm from the eyelid margin, paying special attention to avoid damage to the lashes (Fig. 2 and Fig. 3). In a second pass, the procedure was repeated. After removing the ultrasound gel and eye cover, MGX was done using forceps for meibomian gland (Suzhou Mingren Medical Equipment Co. Ltd., Suzhou, China). In the conjunctival sac, levofloxacin eyedrops was placed. The fellow eye that did not have a chalazion was not treated with IPL or MGX. Same ophthalmologist conducted all IPL treatments and excision operations in outpatient surgery. The patients received separate treatment sessions for 3 weeks, in accordance with the protocols recommended above until the lesion was resolved. The lesions were photographed before IPL treatment and surgery as well as at 1 month after treatment follow-up visit. The chalazion was deemed resolved if the lesion size showed 80–100% reduction and no recurrence after 6 months, based on clinical examination as well as digital photos. Patients whose lesions minimally regressed with IPL or recurred after three sessions were offered steroid injections or surgical incision and curettage.

### Outcome measures

Before treatment, patient histories were taken including age, gender, duration of the chalazia prior to treatment, number of chalazion, the number of previous incision with curettage surgery and eyelid margin abnormality of vascular engorgement. The diameter (mm) of each chalazion was measured with a ruler, and the sum of each chalazion in horizontal size was defined as the total size. Eyelid margin vascularity, graded as 0 = none; 1 = mild; 2 = moderate; 3 = severe engorgement^19^. For evaluation of treatment efficacy, the following parameters were measured before and at 1 month after the final IPL-MGX treatment. The characteristics of the chalazion, including its location and size, hyperemia, and meibum quantity and grade were determined using slit-lamp microscopy. Keratograph 5M (Oculus, Wetzlar, Germany) was utilized to measure noninvasive tear film breakup time (NIBUT), tear meniscus height (TMH), and bulbar conjunctival hyperemia in an automated, non-invasive and objective way according to the manufacturer's instructions^20^. The R-Scan system allowed to perform automatic classification of conjunctival hyperemia. The software autodetected the bulbar and limbal conjunctiva while the eye looking straight ahead and quantified bulbar (nasal, temporal, and overall) and limbal (nasal and temporal) redness grades on a 0 to 4 point scale in 0.1 increments. Noninvasive meibography analyzed meibomian gland morphology. Meibomian gland dropout was divided into four grades: grade 0, no loss; grade 1, less than one-third loss; grade 2, one-third to two-thirds loss; and grade 3, two-thirds or more loss. The higher the score, the greater the meibomian gland dropout area^21^.

Meibomian gland function was examined according to International Workshop on Meibomian Gland Dysfunction standards^22^. Quality of expressed meibum: 1, clear; 2, cloudy; 3, granular, and 4, toothpaste. The higher the score, the worse the quality of the meibum. The central five glands of the lower eyelids were pushed using a meibomian gland evaluator (Tear Science, Inc., Milpitas, USA) to determine expressibility^23^. The scoring criteria: 0, all glands; 1, 3 or 4; 2, 1 or 2; and 3, no glands expressible.

### Safety analysis

All adverse events were documented at every planned and unscheduled research visit. Before and 1 month after the last treatment, eyelid skin burns, redness or swelling, visual acuity, lens opacity, intraocular pressure, as well as fundus examinations were done.

### Statistical analysis

Only the treated eyes were included in all efficacy analyses. Normality and variance homogeneity of data were analyzed by using Shapiro–Wilk normality test and Levene’s test for homogeneity of variances. All data are expressed as mean values and standard deviation (mean ± standard deviation). The Wilcoxon signed-rank test was used to compare the chalazion size, surgery times, TMHs, NIBUTs, bulbar conjunctival hyperemia scores, meibum scores, visual acuities, and intraocular pressures before and after treatment. The Mann–Whitney U test was performed to compare quantitative variables between the IPL-MGX and Control groups. Sex and chalazion location, onset, and recurrence rates were compared using a *χ*^2^ test. All statistical analyses were performed using SPSS 26.0 (SPSS, IBM Corporation, Chicago, IL, USA). *P* values < 0.05 were regarded as statistically significant for all two-sided statistical tests.

**Ethics approval The study was approved by the** Institutional Review Board of the Affiliated Second Hospital of Zhejiang University.

## Results

Ninety-five eyes of 64 patients were treated in the IPL-MGX group, and 95 eyes of 72 patients were treated in the Control group. Most of the patients were referred with recurrent-onset chalazion. 85.3% of the cases in the IPL-MGX group and 70.5% of the cases in the Control group were primary or secondary recurrence after a previous episode. Before treatment, IPL-MGX mean lesion duration was 3.4 weeks and Control were 3.7 weeks. The prevalence rate of eyelid margin vascular engorgement was 36.8% in the IPL-MGX group and 32.6% in the Control group. The mean total size of the chalazia in the IPL-MGX group was 1.0 ± 0.8 cm, with previous excisions times 0.8 ± 0.9. (Table 1).

**Table 1:** Characteristics of the study subjects in the IPL-MGX and Control groups.

Characteristic	IPL-MGX group	Control group	*P* value
No. Eyes (patients)	95 (64)	95 (72)	–
Gender			0.07
Male	26 (40.6%)	35 (48.6%)	–
Female	38 (59.4%)	37 (51.4%)	–
Age (years) (± SD; range)	34.9 ± 9.0 (19–57)	37.4 ± 10.7 (19–67)	0.06
Duration of chalazion (week)	3.4 ± 1.9	3.7 ± 1.8	0.23
Location			0.24
RUL	21 (22.1%)	17 (17.9%)	–
RLL	12 (12.6%)	20 (21.1%)	–
RUL + RLL	16 (16.8%)	13 (13.7%)	–
LUL	19 (20%)	22 (23.1%)	–
LLL	13 (13.7%)	17 (17.9%)	–
LUL + LLL	14 (14.8%)	6 (6.3%)	–
Number of chalazion			0.16
1	38 (40%)	45 (47.3%)	–
2	30 (31.6%)	25 (26.3%)	–
3	10 (10.5%)	16 (16.9%)	–
4	8 (8.4%)	6 (6.3%)	–
5	4 (4.2%)	3(3.2%)	–
6	5 (5.3%)	0 (0%)	–
Onset			0.13
New	14 (14.7%)	28 (29.5%)	–
Recurrent	81 (85.3%)	67 (70.5%)	–
Total size (± SD; cm)	1.0 ± 0.8	0.9 ± 0.6	0.6
Number of previous excision surgery	0.8 ± 0.9	0.7 ± 0.8	0.47
Lid margin vascular engorgement	35 (36.8%)	31 (32.6%)	0.44

RUL, right upper eyelid; RLL, right lower eyelid; LUL, left upper eyelid; LLL, left lower eyelid; SD = standard deviation. *P values < 0.05 were considered significant. Sex and chalazion location, onset, and recurrence rates were compared using a χ2 test. The Wilcoxon signed-rank test was used to compare the chalazion size, surgery times.

Most patients (66 eyes; 69.5%) had 4 rounds of IPL-MGX treatment, with others receiving 3 rounds (16 eyes; 16.8%), 5 rounds (11 eyes; 11.6%), or 6 rounds (2 eyes; 2.1%; Table 2). The mean number of IPL-MGX treatments was 4.0 ± 0.61 (range, 3–6). The mean number of surgical excisions and drainages in the Control group was 2.1 ± 0.7 (range, 1–4). The total size of the chalazia in the IPL-MGX group was decreased significantly from 1.0 ± 0.8 to 0.4 ± 0.6 cm after the final treatment, whereas that in the control group was significantly reduced from 0.9 ± 0.6 to 0.2 ± 0.5 cm. After the final treatment, the total size of the chalazia in the IPL-MGX group and Control group showed a significant decrease compared with that at baseline (*P* < 0.001). Moreover, resolution of the chalazion was achieved in 67 eyes (70.5%) in the IPL-MGX group and in 73 eyes (76.8%) in the Control group (*P* = 0.323). The patients with recurrent lesions (14 eyes, 14.7%) in the IPL-MGX group that failed to respond to 2 courses IPL-MGX therapy were recommended to undergo steroid injection or incision with curettage. The greatest advantage of IPL-MGX was the significantly reduced chalazion recurrence compared to the Control group (14 eyes, 14.7% vs 36 eyes, 37.9%; *P* < 0.001).

**Table 2:** Characteristics of the IPL-MGX and Control groups 1 month after the final of treatment and follow-up visits.

	IPL-MGX Group	Control Group	*P* value
Number of IPL			
3	16 (16.8%)	–	–
4	66 (69.5%)	–	–
5	11 (11.6%)	–	–
6	2 (2.1%)	–	–
Location			0.25
RUL	14 (21.8%)	4 (14.8%)	–
RUL + RLL	12 (18.8%)	3 (11.2%)	–
LUL	9 (14.1%)	5 (18.5%)	–
LLL	6 (9.4%)	7 (25.9%)	–
LUL + LLL	13 (20.3%)	1 (3.7%)	–
Total size (± SD; cm)	0.4 ± 0.6	0.2 ± 0.5	< 0.001***
Resolution rate	70.5%	76.8%	0.323
Average number of IPL (± SD)	4.0 ± 0.61	–	–
Average number of excision (± SD)	–	2.1 ± 0.7	–
Chalazion recurrence	14 (14.7%)	36 (37.9%)	< 0.001***
Post-treatment			
Steroid injection	11 (78.6%)	32 (88.9%)	–
Incision with curettage	3 (21.4%)	4 (11.1%)	–

RUL, right upper eyelid; RLL, right lower eyelid; LUL, left upper eyelid; LLL, left lower eyelid; SD = standard deviation. *P values < 0.05 were considered significant (The Mann–Whitney U test).

**Figure 4:**
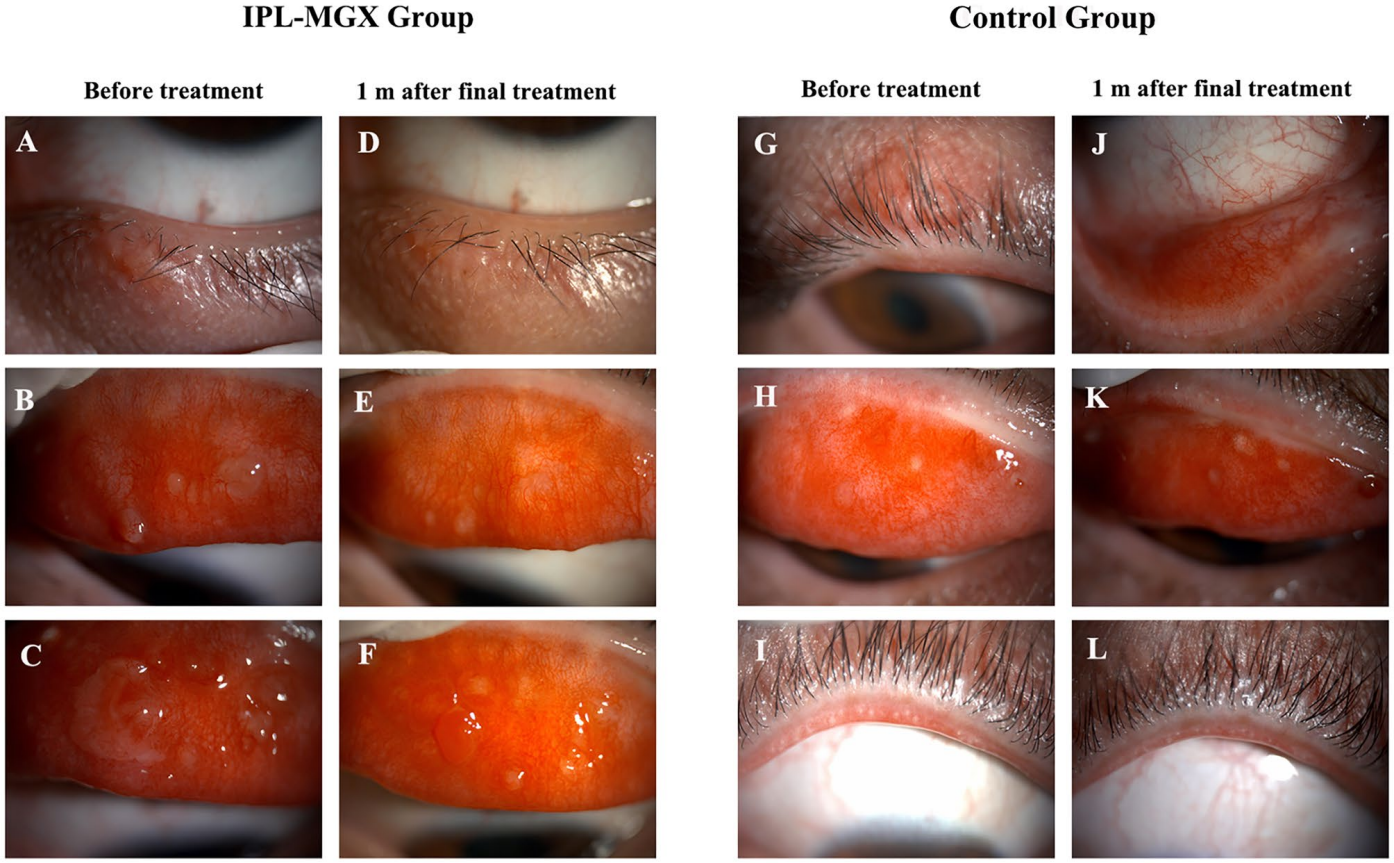
(**A–F**) Case 25 in the IPL-MGX group exhibited considerable improvement in the chalazion size from baseline to 1 month after final treatment. (**G–L**) A patient in the Control group showed a recurrent chalazia in the lower eyelid and no obvious improvement in lid margin vascular engorgement.

**Figure 5:**
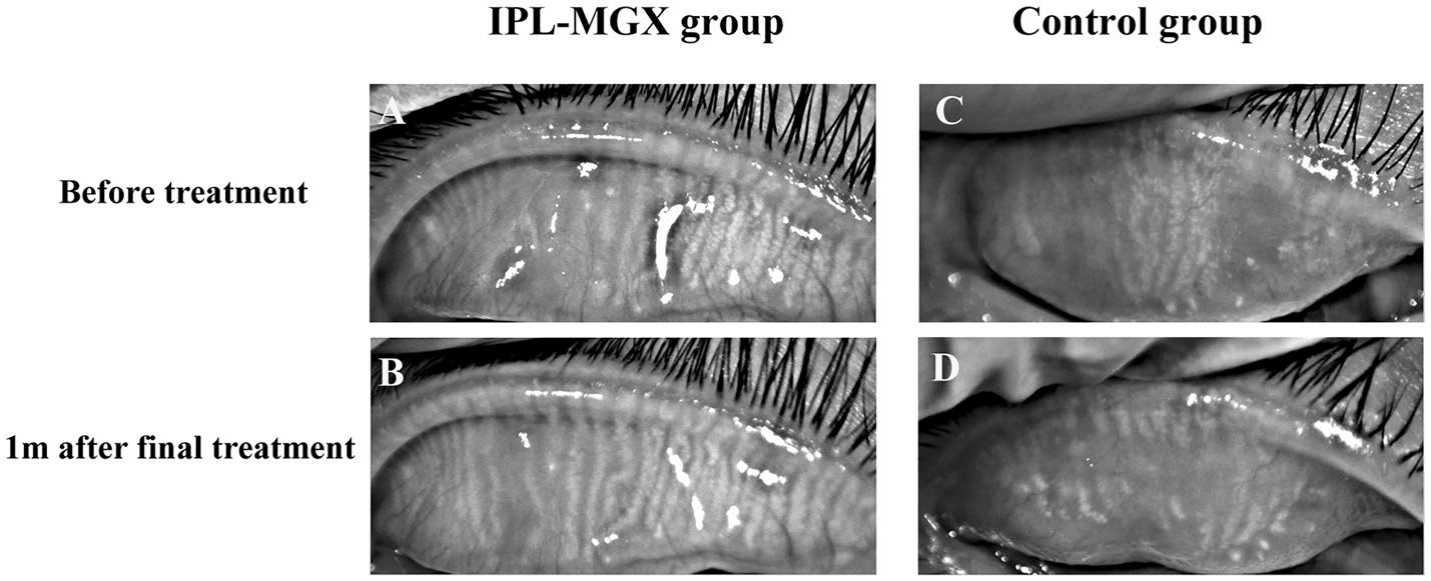
Representative infrared meibography images from the superior eyelids of IPL-MGX (**A**, **B**) and Control (**C**, **D**) participant from baseline to 1 month after final treatment. Overall, the Control participant demonstrates more extensive meibomian gland dropout than the IPL-MGX participant.

**Figure 6:**
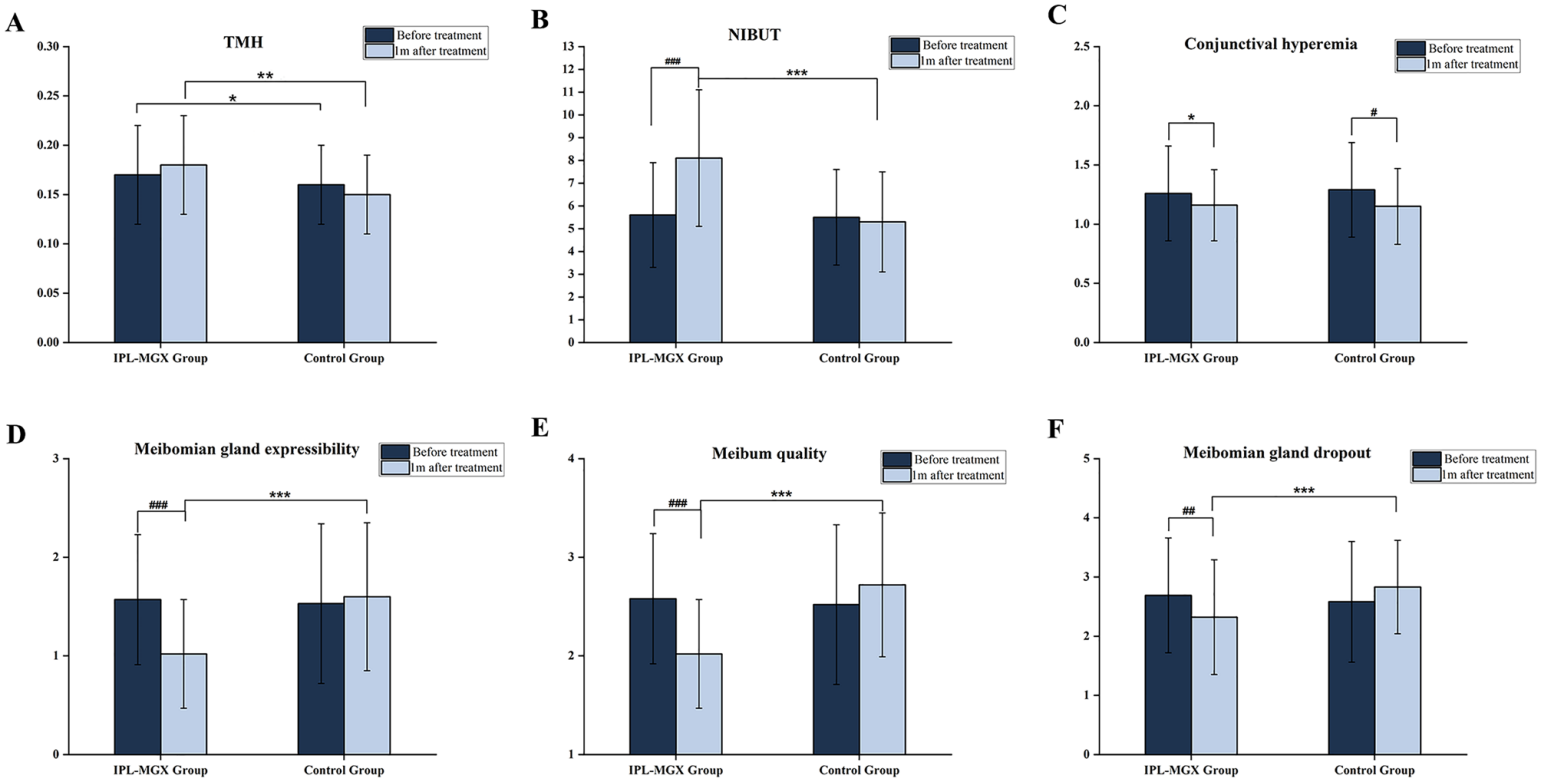
(**A**-**F**) Changes in the TMH, NIBUT, conjunctival hyperemia, the quality and expressibility of meibum, meibomian gland dropout on before and 1 month after the final treatment session in IPL-MGX and Control groups. (Data are shown as mean ± SD, IPL-MGX group versus Control group, *P < 0.05, ** *P* < 0.01, *** *P* < 0.001; before treatment versus 1 month after the final treatment, #*P* < 0.05, ## *P* < 0.01, ### *P* < 0.001, Wilcoxon signed-rank test).

Before treatment, there were no significant differences in NIBUT, conjunctival hyperemia, or meibum parameters between the two groups except TMH. TMH was lower in the Control group than in the IPL-MGX group (*P* = 0.045). The IPL-MGX group's mean TMH score rose 1 month after final treatment compared to the Control group (0.18 ± 0.05 vs. 0.15 ± 0.04; *P* = 0.003). However, the TMH score did not significantly differ between before and after treatment in both groups. The NIBUT was significantly prolonged from 5.6 ± 2.3 s to 8.1 ± 3.0 s at 1 month after the last treatment of IPL-MGX (*P* < 0.001). After treatment, the IPL-MGX group showed statistically significant increases in NIBUT and statistically significant reductions in meibum grade compared to the Control group. After the final treatment, the IPL-MGX group showed considerable improvements in NIBUT, meibum grade, and meibomian gland dropout (Figs. 4, 5, 6). However, only bulbar conjunctival hyperemia statistically significantly decreased in the control group (*P* = 0.011; Fig. 6; Table 3).

**Table 3:** Changes treatment measures in IPL-MGX and Control groups at visit before and 1 month after final treatment.

	Before treatment	1 month after final treatment	*P* value
TMH			
IPL-MGX group	0.17 ± 0.05	0.18 ± 0.05	0.253
Control group	0.16 ± 0.04	0.15 ± 0.04	0.83
*P* value	0.045*	0.003**	
NIBUT			
IPL-MGX group	5.6 ± 2.3	8.1 ± 3.0	< 0.001###
Control group	5.5 ± 2.1	5.3 ± 2.2	0.41
*P* value	0.769	< 0.001***	
Conjunctival hyperemia			
IPL-MGX group	1.26 ± 0.4	1.16 ± 0.3	0.051
Control group	1.29 ± 0.4	1.15 ± 0.32	0.011#
*P* value	0.655	0.73	
Meibomian gland expressibility			
IPL-MGX group	1.57 ± 0.66	1.02 ± 0.55	< 0.001###
Control group	1.53 ± 0.81	1.6 ± 0.75	0.56
*P* value	0.68	< 0.001***	
Meibomian gland quality			
IPL-MGX group	2.58 ± 0.66	2.02 ± 0.55	< 0.001###
Control group	2.52 ± 0.81	2.72 ± 0.73	0.91
*P* value	0.087	< 0.001***	
Meibomian gland dropout			
IPL-MGX group	2.69 ± 0.97	2.32 ± 0.97	0.003##
Control group	2.58 ± 1.02	2.83 ± 0.79	0.07
*P* value	0.533	< 0.001***	

*P values < 0.05 (The Mann–Whitney U test) and #*P* values < 0.05 (The Wilcoxon signed-rank test) were considered significant.

Of the 95 study patients who received IPL treatment, 38 individuals complained of minor pain and burning sensation during the treatment, and mild redness of the eyelids was observed immediately after the IPL treatment. However, none of these patients dropped out of the study due to discomfort. All 38 patients experienced relief from their symptoms following the 5 min of cold compress application. No irreversible eyelid skin injury and loss of eyelashes occurred. Visual acuity and intraocular pressure showed no significant changes after both treatments (all *P* > 0.05; Table 4). No significant changes in the anterior segment, lens opacity, and fundus condition were observed in all patients.

**Table 4:** A comparison of visual acuity and intraocular pressure before and 1 month after the final treatment.

	Before treatment	1 month after final treatment	*P* value
IPL-MGX group			
Visual acuity	0.9 ± 0.2	0.9 ± 0.2	0.54
Intraocular pressure (mmHg)	13.2 ± 3.9	13.5 ± 3.1	0.15
Control group			
Visual acuity	0.8 ± 0.2	0.8 ± 0.2	0.39
Intraocular pressure (mmHg)	13.0 ± 3.2	12.8 ± 2.9	1.0

^#^*P* values < 0.05 were considered significant (The Wilcoxon signed-rank test).

## Discussion

It is a prospective and controlled study to show that IPL therapy with a light-guided tip administered instantly to the eyelids and chalazion region significantly enhanced objective symptoms as well as signs in comparison to conservative treatment for participants with chalazion. Our results showed that light-guided-tip IPL combined with MGX therapy resulted in a comparable resolution rate in chalazion with excision surgery, decrease in chalazion recurrence rate, increase tear breakup time, and better meibomian gland function. This study obtained a new insight into the effect of IPL therapy applied directly on the eyelid on chalazion resolution.

Previous studies reported that the success rate of conservative therapy ranges widely from 25 to 87% and was greatly dependent on patient education by the physician and patient compliance with the treatment regimen^24,25^. The range of the success rate was 8.7 to 86.7% for 1 steroid injections. The success rate for the second steroid injections was 19.0%, with a range of 0–53.8%^26^. The recurrence rate ranged from 0 to 27.3% for steroid injection^27,28^. We previously reported a recurrence rate of 45.6% for recurrent chalaziosis^7^. In this study we found that IPL-MGX therapy resulted in significant resolution or nearly resolution of primary and recurrent chalazia after an average of 4 sessions in more than 70% of cases. The IPL-MGX therapy showed a similar clinical remission response as excision surgery but a much lower recurrence rate. Another study reported a higher resolution rate of 96.15% after 1–3 IPL sessions in the acute inflammatory phase of chalazion regardless of the duration and recurrence of the lesion^29^. Arita et al. evaluated the efficacy of IPL on cheeks and upper and lower eyelids, combined with MGX, for the treatment of refractory multiple and recurrent chalazia without surgery or curettage. They suggested that the symptoms, size of chalazion, number of Demodex mites, and lid margin abnormalities significantly improved after IPL-MGX^16^. A concern raised regarding the efficacy of IPL is that in the early acute inflammatory phase, the chalazion may show a better response to conservative treatment or incision and curettage. This study supports our suggestions that IPL given directly on the eyelid may be a suitable alternative to excision surgery for primary or recurrent chalazion with inflammation^7^.

IPL therapy was applied directly to both the upper as well as lower eyelids of individuals diagnosed with MGD by Bei Rong et al.^30^ Meibomian gland secretion score, tear film break-up time, cornea fluorescein staining score along with dry eye evaluation score were significantly improved in the eyes with MGD. In our study, IPL-MGX treatment significantly improved the NIBUT, conjunctival hyperemia score, and meibomian gland condition in terms of, for example, meiboscore, meibum grade, and meibomian gland dropout at 1 month after the final IPL treatment session. The NIBUT, meibum quality and expressibility scores, and meibomian gland dropout were significantly improved at 1 month after the final IPL-MGX treatment compared with before treatment. Meibomian gland dropout, which is thought to be an possibly irreversible result of chalazion incision surgery, was ameliorated by IPL therapy. In addition, TMH, NIBUT, and meiboscore were significantly improved after treatment in the IPL-MGX group compared with the Control group.

IPL is commonly applied on facial skin adjacent to the lower eyelid^31,32^ to ensure treatment safety, but we applied IPL directly on the eyelid, using an eye shield to protect the conjunctival sac. No severe adverse events were observed during the study. Approximately one-third of the patients complained of slight burning sensation and indicated mild erythema on the eyelid due to the two-step IPL treatment. This suggests the need for a moderated and optimized treatment procedure.

Several studies on IPL treatment have reported that a positive feedback loop underlies the inflammatory process that is disrupted by IPL through upregulating anti-inflammatory agents and/or downregulating proinflammatory agents^33–35^. Second, the temperature of the skin can rise up to almost 70 °C during IPL therapy. A thermal response unclogs meibomian glands, liquefies meibum, and improves distribution of meibomian gland lipids over the ocular surface^36,37^. Moreover, *Demodex folliculum,* a potential mediator of blepharitis, whose exoskeleton contains pigmented chromophores that absorb IPL^38^. Demodex eradication reduces the microbial load, which contributes to chronic eyelid inflammation^39^. In our study, we found that IPL-MGX treatment improved meibum quality, meibum secretion function and meibomian gland dropout, whereas conservative treatment or excision surgery alone did not.

Conservative treatment measures for chalazion include warm compress, appropriate eyelid hygiene, and antibiotic ointment therapy. Only one-fourth of chalazion cases may resolve spontaneously within an average duration of 6 months^24^. The efficacy of steroid injections for chalazia have been evaluated in prior trials. Khurana et al. reported that intralesional therapy of steroid injection is equally effective for small, marginal and multiple chalazia^41^. Serious complications of steroid injections, including skin depigmentation, fundus vascular occlusion, post-injection hemorrhage and inadvertent globe penetration, are rare^42–45^. Surgical incision may be suitable for large or infected chalazion or lesions that do not respond to conservative treatment^46^. However, permanent eyelid dysfunction and aesthetic flaws may arise from eyelid marginal and numerous incisions for recurring chalazion.

Overall, our patients were satisfied with the light-guided-tip IPL procedure. In the majority of instances, they preferred repeated IPL treatment to surgery of incision. The benefits of IPL therapy include the simplicity and repeatability of the procedure; its abilities to treat patients who could not tolerate long and multiple surgeries, decrease the recurrence rate of refractory chalazion, and treat lesions near the lacrimal punctum; and its use as an alternative to surgery in cases of multiple small and marginal chalazia with inflammation, where surgery may result in permanent functional and aesthetic defects. A repeated IPL treatment would be accepted by patients as opposed to more surgeries. As IPL could improve chalazion lesions by closing the abnormal blood vessels and preventing the secretion of inflammatory mediators, on the basis of the results of the present study. We propose that IPL therapy may be useful for treating such lesions during the acute inflammatory stage and/or in the existence of superficial vessels. The use of IPL is also recommended for the treatment of multiple and marginal chalazia. However, for infected lesions, chronic inflammation, and larger lesions, treatment with incision and curettage has more favorable outcomes.

The limitations of study include the potential bias due to the ophthalmologists' varied techniques and previous surgical and examination experiences with initial chalazia. To minimize these variations, a single ophthalmologist was chosen to evaluate the characteristics of the chalazia and perform IPL. Moreover, the number of IPL-MGX treatments sessions varies depending on the size, age, and length of the chalazion, as well as the patient's level of comfort with the procedure. In future studies, a larger sample size of patients with chalazion and a more controlled prospective random experimental design will be preferred. In subsequent research, expanded follow-up, modifying the IPL therapy settings for the light guide tip to optimize the therapeutic impact, and investigating the underlying processes of IPL's effects on chalazion are all recommended.

In conclusion, repeated light-guided-tip IPL treatment with MGX is effective and safe for decreasing the size and lowering the recurrence rate of chalazion and promoting meibomian gland function improvement. The findings suggest a novel technique for treating recurrent or refractory MGD-related chalazia. Further studies are required to determine if light-guided-tip IPL treatment would improve the outcomes of specific types of refractory chalazion in when used in clinical settings.

## Data Availability

The datasets used and analyzed during the current study available from the corresponding author on reasonable request.
